# A case–control study of risk factors for HIV-negative children with cryptococcal meningitis in Shi Jiazhuang, China

**DOI:** 10.1186/1471-2334-12-376

**Published:** 2012-12-26

**Authors:** Jianhua Guo, Jikun Zhou, Shiyong Zhang, Xin Zhang, Jing Li, Yinqi Sun, Shunxiang Qi

**Affiliations:** 1 Shi Jiazhuang Center for Disease Prevention and Control (CDC), Shi Jiazhuang 050011, People’s Republic of China; 2 Hebei Provincial children's hospital, Shi Jiazhuang 050019, People’s Republic of China; 3 Hebei Provincial Center for Disease Prevention and Control (CDC), Shi Jiazhuang 050011, People’s Republic of China; 4 Institute for Epidemiology and Health Emergency, Shi Jiazhuang Center for Disease Prevention and Control (CDC), No.3 Li Kang Street, Shi Jiazhuang 050011, China

**Keywords:** Cryptococcosis, Cryptococcal meningitis, Epidemiologic characteristics, Clinical features, Bird droppings and sacrophytes, Risk factors, Children

## Abstract

**Background:**

Although cryptococcal meningitis (CM) is an emerging disease worldwide, there have been few studies of the characteristics and risk factors of CM in children.

**Methods:**

We used data collected from May 2007 through April 2012 in the Acute Meningitis-Encephalitis Syndrome Surveillance project in Shi Jiazhuang, China to describe the epidemiologic, clinical, and laboratory findings in children with CM. Furthermore, a matched case–control study was used to determine risk factors of CM.

**Results:**

Overall 23 HIV-negative children with CM (median age: 10.91 years; range: 5 months-17 years) were enrolled in our study. The average annual incidence of CM was 0.43/100,000 with a fatality rate of 1.7%. Most patients were males (60.87%) and rural children (73.91%). Common clinical symptoms included increased intracranial pressure, such as headaches (78.3%), nausea (60.9%), altered mental status (56.5%), vomiting (52.2%), and seizures (43.5%), and frequent laboratory findings consisted of blood leukocytosis (87.0%), decreased CSF glucose (87.0%), pleocytosis (82.6%), increased intracranial pressure (73.9%) and elevated CSF proteins (65.2%). Epidemiologic, clinical, and laboratory findings were similar between patients with and without underlying diseases. Multivariate logistic regression analysis showed that children who had contact with birds/bird droppings or saprophytes were more likely to be infected than those without such contact (odds ratio(OR) =11.82; 95% confidence interval (CI), 2.21-62.24; *P* = 0.004). Patients with an interval of ≥20 days from onset to admission were at high risk for CM (OR= 5.31; 95%CI, 1.58-17.89; *P* = 0.007).

**Conclusions:**

Our findings show that CM is an uncommon disease with a high mortality rate in children. Although additional studies are needed to find effective prevention and treatments for CM, clinicians should consider CM as a potential cause for pediatric meningitis in children, particularly boys from rural areas, who had contact with birds/bird droppings or saprophytes and in children who did not receive prompt medical attention.

## Background

Cryptococcosis is an emerging disease arising due to the global HIV/AIDS pandemic. *Cryptococcus neoformans* is the most common cause of cryptococcosis worldwide. Cryptococcosis is responsible for cryptococcal meningitis (CM), the most common fungal infection of the central nervous system and a significant cause of morbidity and mortality among patients with HIV/AIDS [1]. Few studies of pediatric CM have been reported, but CM is believed to be less frequent in children than in adults, regardless of immune status [2]. Recently, higher numbers of CM cases in children have been reported worldwide [3-13]. However, these reports come from small case studies, literature reviews, or studies comprised mostly of adults. In addition, the epidemiologic characteristics, clinical features, laboratory findings, and outcomes for pediatric patients are inconsistent among the different studies. Data on the overall incidence of children with CM are scarce, and understanding which children are at highest risk of CM is lacking.

Here, we analyzed the epidemiologic characteristics, clinical features, laboratory findings, outcomes, and risk factors for CM in pediatric patients without HIV/AIDS. Study subjects were identified by the Acute Meningitis and Encephalitis Syndrome (AMES) surveillance project in Shi Jiazhuang, China.

## Methods

### Data source and study population

Data for this study were obtained from the Acute Meningitis and Encephalitis Syndrome (AMES) surveillance database. This database contains demographic information, clinical data, laboratory findings, and outcome for AMES patients; trained individuals entered these data into EPIDATA databases ( http://www.epidata.dk/history.htm) with the function of Double Entry and Validation.

Surveillance was conducted for the AMES project in 44 referral hospitals with an aggregate population of around 5.2 million from May 2007 through April 2012, in Shi Jiazhuang. The population in Shijiazhuang was stable during the study period. Children < 18 years old comprise 20.8% of the total population. Patients with AMES traditionally seek medical care at the referral hospitals rather than at community hospitals and private clinics. Member hospitals receive most of the patients across Shi Jiazhuang. An AMES case was defined as a patient with acute onset of fever ≥38°C, changes in mental status, and/or meningeal signs. Additionally, an initial diagnosis in member hospitals with Japanese encephalitis, viral encephalitis, viral meningitis, viral meningoencephalitis, other encephalitis, meningococcal meningitis, purulent meningitis, cerebrospinal meningitis, tuberculosis meningitis, tuberculosis meningoencephalitis, and other meningitis also qualified as an AMES case. AMES cases were reported to Shi Jiazhuang CDC by clinicians in hospitals. Trained CDC staff performed interviews. To ensure all AMES cases were found, audits were performed every 10 days in each hospital.

Clinicians collected blood (≥ 3 mL) and/or cerebrospinal fluid (CSF) samples from AMES cases with written informed consent have been obtained from their parents in the case of children younger than 18 years. Routine blood test, CSF test, or culture was conducted at each hospital laboratory. Periodic laboratory audits were performed to ensure case ascertainment in each laboratory. Confirmed AMES case was defined as any AMES patient with a positive pathogen identified by culture from CSF and/or blood. A CM case was defined as an AMES patient with positive cryptococcal culture from CSF and/or blood. Cryptococcal cultures were identified by L-canavanine glycine bromothymol blue agar. *C. neoformans* fails to cause a color change on the agar, while the color of the agar changes to blue from green in the presence of *C. gattii*.

### Estimates of risk factors

A matched retrospective case–control study was conducted in a cohort of patients with confirmed AMES to identify risk factors for CM. The case was defined as any patient with CM. The control was defined as any confirmed AMES patient due to another pathogen. All cases and controls were obtained from the AMES database. Each case was matched to four controls, with onset of meningitis occurring within three months of each other. The following data were used to examine the risk factors: gender, age, geographic location, exposure to birds/bird droppings or saprophytes 3 months before illness onset, underlying disease, date of onset of illness, and date of admission.

### Data analysis

Continuous variables were summarized as medians with ranges. Categorical variables were summarized as proportions. Fisher's exact test was used to compare the categorical variable between patients without underlying diseases and those with underlying diseases. Univariate conditional logistic regression models were used to examine gender, age, geographic location, underlying diseases, contact history of birds/bird droppings or saprophytes, and the time interval from illness onset to hospital admission. All variables with a P value <0.10 upon univariate analysis were included in a multivariate conditional logistic regression model to evaluate their independent effects. Backward logistic regression was conducted by removing variables with P > 0.10 upon multivariate conditional logistic regression. Statistical significance was defined as P < 0.05. Data were analyzed using the Stata version 9.0 statistical software (StataPress, College Station, TX, USA).

### Ethical considerations

The Ethics Committee of Shi Jiazhuang CDC and ethical review committees of each participating hospital approved this study.

## Results

### Epidemiologic characteristics

Over a 5-year study period, 23 cases of pediatric CM (Median age: 10.91 years; Range: 5 months-17 years) due to *C. neoformans* were confirmed from positive CSF cultures of HIV-negative children. The average annual incidence and fatality rate of CM was found to be 0.43/100,000 and 1.74% among children < 18 years old. Most of the patients identified were males (60.87%) and from rural environments (73.91%). About half of the patients (43.48%) emerged in June-August during the 5-year period. Most cases (78.26%) had contact with birds/bird droppings or saprophytes before illness onset and 26.1% of the cases had underlying diseases (1 patients with hepatitis B, 1 with renal disease, 1 with diabetes, 1 with aplastic anemia, 1 with tuberculosis, and 1 with hand-foot-mouth disease). None of the patients had traveled outside his/her district for at least one year before becoming ill. Epidemiologic characteristics of patients with or without underlying diseases did not differ (Table 1).

**Table 1:** Characteristics of pediatric patients with cryptococcal meningitis

**Characteristic**	**Patients without underlying diseases (%) (n = 17)**	Patients with underlying diseases* (%) (n = 6)	**Total [no.(%)] (n = 23)**	***P *value**
**Gender**				
Male	11 (78.6)	3 (50.0)	14 (78.6)	0.643
**Age, years**				0.835
0-5	3 (17.6)	1 (16.7)	4 (17.4)	
5-10	5 (29.4)	1 (16.7)	6 (26.1)	
10-15	7 (41.2)	2 (33.3)	9 (39.1)	
15-17	2 (11.8)	2 (33.3)	4 (17.4)	
**Geographic location**				
Rural area	13 (76.5)	4 (66.7)	17 (73.9)	0.632
**Date of illness onset**				0.444
December-February	5(29.4)	0(0.0)	5 (21.7)	
March-May	3(17.6)	1(16.7)	4 (17.4)	
June-August	7(41.2)	3(50.0)	10 (43.5)	
September-November	2(11.8)	2(33.3)	4 (17.4)	
**Contact with birds/droppings or saprophytes**†	12 (70.6)	6 (100.0)	18 (78.3)	0.274
**Time from illness onset to hospitalization (days)**				0.093
1-10	2 (11.8)	1 (16.7)	3(13.0)	
10-20	2 (11.8)	1 (16.7)	3 (13.0)	
20-30	11 (78.6)	1 (16.7)	12 (52.2)	
≥30	2 (11.8)	3 (66.7)	5 (21.7)	
**Clinical symptoms and signs**				
Fever (≥39°C)	8 (47.1)	5 (83.3)	13 (56.5)	0.179
Headache	13 (76.5)	5 (83.3)	18 (78.3)	1.000
Nausea	9 (52.9)	5 (83.3)	14 (78.6)	0.340
Altered mental status	10 (58.8)	3 (50.0)	13 (56.5)	1.000
Vomiting	10 (58.8)	2 (33.3)	12 (52.2)	0.371
Seizures	6 (35.3)	4 (66.7)	10 (43.5)	0.341
Meningeal signs	5 (29.4)	3 (50.0)	8 (34.8)	0.621
Neck stiffness	6 (35.3)	1 (16.7)	7 (30.4)	0.621
Hearing damage	3 (17.6)	1 (16.7)	4 (17.4)	1.000
Visual damage	3 (17.6)	0 (0.0)	3 (13.0)	0.539
Bregmatic eminence	1 (5.9)	0 (0.0)	1 (4.3)	1.000
**Laboratory findings**				
Blood leukocytosis (≥15 × 10^9^/L)	15 (65.2)	5 (83.3)	20 (87.0)	1.000
Decreased CSF glucose (<3.1 mmol/L)	16 (94.1)	4 (66.7)	20 (87.0)	0.155
Pleocytosis (×10^6^/L)				0.483
<10	4 (23.5)	0 (0.0)	4 (17.4)	
10-100	7 (41.2)	2 (33.3)	9 (39.1)	
≥100	6 (35.3)	4 (66.7)	10 (43.5)	
Elevated CSF proteins (≥0.45 g/L)	10 (58.8)	5 (83.3)	15 (65.2)	0.369
Intracranial pressure(>250 mm H_2_O)	14(82.4)	3(50.0)	17 (73.9)	0.279

*Underlying diseases included hepatitis B (1 case, 3 controls), renal disease (1 case, 2 controls), diabetes (1 case, 1 control), aplastic anemia (1 cases, 1 controls), tuberculosis and hepatitis B (1 case, 1 control), and hand-foot-mouth disease (1 case, 4 controls).

†Birds included pigeons, chicken, or parrots; saprophytes included soils, grassland, or rotten vegetables and fruits.

### Clinical features and laboratory findings

The median duration from illness onset to hospital admission was 33 days (range: 2–48 days). The initial diagnoses included meningitis (34.78%), bacterial meningitis (30.43%), viral meningitis (21.74%), and tuberculosis meningitis (13.04%). The first symptoms were actue fever in 78.3% of the patients and headache in 65.2%. Frequent symptoms and signs were fever (100%), headache (78.3%), nausea (60.9%), altered mental status (56.5%), vomiting (52.2%), seizures (43.5%), meningeal signs (34.8%), and stiff neck (30.4%) (Table 1). Blood leukocytosis (87.0%), decreased CSF glucose (87.0%), pleocytosis (82.6%), increased intracranial pressure (73.9%) and elevated CSF proteins (65.2%) were found in most patients (Table 1). Clinical features and laboratory findings did not differ significantly between patients with or without underlying diseases (Table 1).

### Outcome

The median length of hospitalization was 22 days (range: 13–34 days). One patient without underlying disease and another with renal disease died during hospitalization. At discharge, 18 cases had recovered and 3 cases had complications, one with hearing damage, one with mild papilloedema, and one with blurred vision. These 3 patients had recovered by the 6-month follow-up.

### Risk factors

Univariate conditional logistic analysis was used to identify risk factors associated with CM patients in comparison to patients with other types of meningitis (Table 2). When compared to patients < 5 years old, patients ≥ 10 and <15 years old (odds ratio (OR) = 4.36; 95% confidence interval (CI), 1.12-9.81; *P* = 0.033) or patients ≥ 15 and <18 years old (OR = 5.53; 95%CI, 1.19-25.79; *P* = 0.029) were significantly associated with CM. Contact with birds/bird droppings or saprophytes was associated with CM (OR = 9.32; 95%CI, 2.54-34.23; *P* = 0.001). An interval of ≥10 days and <20 days from illness onset to hospital admission (OR = 2.22; 95%CI, 0.35-14.15; *P* = 0.398) was not significantly associated with CM, whereas an interval of ≥20 days from onset to admission was significantly associated with CM (OR = 11.39; 95%CI, 2.20-59.02; *P* = 0.004).

**Table 2:** Univariate conditional logistic analysis of risk factors for cryptococcal meningitis among patients with meningitis and encephalitis

**Factors**	**Case [no.(%)] (n = 13)**	**Control [no.(%)] (n = 52)**	***P value***	**OR (95%CI)**
**Gender**				
Female	9 (21.4)	51 (55.4)	Reference	
Male	14 (78.6)	41 (44.6)	0.190	1.81 (0.75-4.38)
**Age, years**				
0-5	4 (17.4)	36 (39.1)	Reference	
5-10	6 (26.1)	29 (31.5)	0.612	1.51 (0.31-7.35)
10-15	9 (39.1)	21 (22.8)	0.033	4.36 (1.12-9.81)
15-17	4 (17.4)	6 (6.5)	0.029	5.53 (1.19-25.79)
**Geographic location**				
Urban areas	6 (26.1)	32 (34.8)	Reference	
Rural areas	17 (73.9)	60 (65.2)	0.384	1.75 (0.54-5.66)
**Time from illness onset to hospitalization (days)**				
1-10	2 (8.7)	35 (38.0)	Reference	
10-20	3 (13.0)	24 (26.1)	0.398	2.22 (0.35-14.15)
20-30	12 (52.2)	17 (18.5)	0.004	11.39 (2.20-59.02)
≥30	6 (26.1)	16 (17.4)	0.055	5.05 (0.96-26.84)
Contact with birds/droppings or saprophytes*				
No	5 (21.7)	56 (60.9)	Reference	
Yes	18 (78.3)	36 (39.1)	0.001	9.32 (2.54-34.23)
**Underlying disease**†				
No	17 (73.9)	80 (87.0)	Reference	
Yes	6 (26.1)	12 (13.0)	0.160	2.05 (0.75-5.58)

* Birds included pigeons, chicken, or parrots; saprophytes included soils, grassland, or rotten vegetables and fruits.

† Underlying diseases included hepatitis B (1 case, 3 controls), renal disease (1 case, 2 controls), diabetes (1 case, 1 control), aplastic anemia (1 case, 1 control), tuberculosis and hepatitis B(1 case, 1 control), and hand-foot-mouth disease (1 case, 4 controls).

Furthermore, we performed a multivariate conditional logistic regression analysis of risk factors for CM (Table 3). Subjects with a history of contact with birds/bird droppings or saprophytes were more likely to be infected than those without contact (OR = 11.82; 95%CI, 2.21-62.24; *P* = 0.004).

**Table 3:** Multivariate conditional logistic analysis of risk factors for cryptococcal meningitis among patients with meningitis and encephalitis

**Risk Factor**	***P *value**	**OR (95%CI)**
Time from illness onset to hospital admission (≥20 day)	0.007	5.31(1.58-17.89)
Contact with birds/droppings or saprophytes*	0.004	11.82(2.21-62.24)

* Birds included pigeons, chicken, or parrots; saprophytes included soils, grassland, or rotten vegetables and fruits.

Overall, our findings suggest that contact with birds/bird droppings or saprophytes and a longer interval from illness onset to hospital admission are independent risk factors for CM.

## Discussion

Here, we analyzed epidemiology, clinical and laboratory characteristics, and risk factors associated with CM due to *C. neoformans* in HIV-negative children. Similar to the incidence in children of Taiwan with unknown HIV status reported previously [11], we found an average annual incidence of 4.3 cases of CM per million HIV-negative children with a fatality rate of 1.7% in mainland China.

Two species in the genus Cryptococcus, *C. neoformans* and *C. gattii,* can infect humans. The former, which is found commonly worldwide, infects mostly immunocompromised patients [14,15], whereas the latter affects both immunocompetent and immunocompromised individuals [16,17]. In our study, children without underlying diseases comprised 74% of CM patients caused by *C. neoformans*, consistent with other reports from mainland China [18]. This finding may be explained by the genetic homogeneity between Chinese *C. neoformans* strains [18]. However, data demonstrate that *C. gattii,* which was formerly described as a geographically restricted species [19], is spreading at a great rate around the globe. Cases caused by this species have been reported not only from the tropical and subtropical areas, but also from the Pacific coast of Canada and the United States [20]. Moreover, China, Europe, and Japan confirm that this fungus can also occur in temperate climates [18,21,22].

The proportion of immunocompetent patients with CM varies greatly between the HIV-negative patients in different regions and countries [8,23-28], regardless of the age of patients. For example, in Taiwan, about 50% of HIV-negative children with CM exhibited underlying disease [8,23] whereas 38% of CM patients in India had no underlying disease [24]; in Vietnam, 81% of the CM patients had no underlying disease [25], but in the United States, about 70% of HIV-negative adults with CM had underlying disease [26]. Subtle defects in immunity [27] or idiopathic CD4 lymphopenia [28] could account for these variations, as could ethnic differences. Further studies are needed to determine the cause of regional variation in the incidence of CM among immunocompetent populations.

However, the epidemiologic characteristics, clinical features, and laboratory findings associated with CM were similar for children with and without underlying diseases. The median age of infected children was about 12 years old, consistent with prior studies of CM in HIV-negative children [8,9]. Like in other hospital-based reports of CM in children, most of the patients in our study were boys [12,29]. A population-based surveillance in South Africa also showed that boys are significantly more likely to become infected with cryptococcosis than adults, but it did not evaluate the gender distribution for CM in children [30].

Of the children included in our study, 73.9% of the patients were from rural areas, suggesting that there is increased environmental exposure in rural areas. Similar results were obtained in a population-based study in Australia and New Zealand [4], although few HIV-negative children with CM were included in these studies. Furthermore, about half of the CM cases in our study occurred in summer in Shijiazhuang; the reason for this is unclear. Although time distribution is an important epidemiologic characteristic of diseases, the seasonal distribution of CM in children has not been reported. When compared to children with meningitis caused by other pathogens, a conditional logistic analysis showed that age, gender, and geographic location were not risk factors for CM in pediatric patients. We did not test onset of illness as a risk factor, because it was the matching variable in the case–control study. Thus, further studies are needed to confirm whether or not age, gender, geographic location, and onset of illness are risk factors associated with CM in children.

In our study, fever was the most frequent sign of CM, followed by headache, nausea, altered mental status, and vomiting. Hearing and visual damage were less common during the course of the disease. These frequent characteristics were secondary to increased intracranial pressure (ICP) and consistent with the laboratory findings of ICP. Pleocytosis, elevated CSF proteins, and decreased CSF glucose levels were also common. These clinical and laboratory findings are somewhat different from those previously reported in HIV-negative or HIV-positive children [8,12,29]. In HIV-negative children with CM, one study reported a meningeal irritation sign to be the most common manifestation, followed by headache, vomiting, and pleocytosis in 9 HIV-negative children with CM [8]. Yet another study described hearing and visual damage to be common symptoms in 11 HIV-negative children with CM [12]. These different clinical and laboratory findings may be due to different patient populations enrolled in the various studies. The main reason for the difference between HIV-negative or HIV-positive children may be the immune status of the patients. Overall, the characteristics of CM did not differ from those of other forms of meningitis, thus we could confirm the diagnosis of CM only through identification of *Cryptococcus* in the CSF. The difficulty in diagnosis is likely why patients were misdiagnosed with other forms of meningitis prior to CSF culture.

Although the characteristics of CM are indistinguishable from other forms of meningitis, the treatment may differ. Thus, early identification and prevention are critical for management of CM in the pediatric population. Infection with HIV/AIDS is the most common risk factor for CM in adults, followed by other comorbid conditions, such as cancer, organ transplantation, renal disease, and connective tissue disorders. Furthermore, a population-based multistate study revealed smoking and outdoor occupations are associated with an increased risk of cryptococcosis in adults infected with HIV [3]. However, the risk factors associated with CM in children have not been defined. Our study shows that contact with birds/bird droppings or saprophytes and a longer interval between onset of illness and hospital admission are independent risk factors for CM, but not for other types of meningitis in children. In contrast to children with meningitis due to other pathogens, underlying disease in children with CM is not a significant risk factor. Thus, our findings suggest that contact with birds/bird droppings or saprophytes and a longer interval between onset of illness and hospital admission may be used to distinguish CM from other types of meningitis in pediatric patients.

Although our observations provide valuable insight into pediatric CM, they are not without limitations. The incidence of HIV-negative children with CM was likely underestimated because only fungal culture was used to identify *C. neoformans*. The traditional diagnostic techniques used to identify this infection include culture analysis, India ink staining of CSF, and cryptococcal polysaccharide antigen detection in the serum and CSF. Lateral flow immunoassay is a new diagnostic method for cryptococcosis, and it shows comparable results to the widely approved enzyme immunoassay for cryptococcal antigen [31]. Although every one of these methods can be used to detect cryptococcosis, a combination of methods may detect more cases with incidences closer to those reported for HIV-negative children with CM in Shi Jiazhuang. Additionally, the sample size used in this study may have limited the power to detect risk factors although our results are statistically significant and support our conclusion that exposure to birds/bird droppings or saprophytes and the interval from illness onset to hospital admission are independent risk factors for CM. However, a larger sample size may allow detection of additional risk factors. Continuous surveillance or a multicenter study would need to be conducted to detect more cases that could be used to assess for additional risk factors. Furthermore, although half of the CM cases emerged in summer, this case–control study lacks a matched variable that would have allowed us to determine if there is a seasonal risk of developing pediatric CM.

Overall, our findings suggest that we should reconsider strategies for managing and preventing CM in children. Instead of simply administering drug prophylaxis, a primary, secondary, and tertiary strategy for prevention and control of CM could be tailored to individuals at high risk for CM. For instance, our results suggest that children should avoid contact with birds/bird droppings and saprophytes, sick children should receive medical care as soon as possible, and clinicians need to consider CM as a potential cause for meningitis especially in boys and in children, who had contact with birds/bird droppings or saprophytes and in children who did not receive prompt medical attention. Furthermore, public health studies are needed to identify and/or develop effective antifungal drugs, vaccines [32], and screening strategies [33].

## Conclusions

In summary, we found that although CM is not common in HIV-negative children, the mortality rate associated with CM is high in HIV-negative children. The epidemiology and clinical and laboratory characteristics of CM do not differ between HIV-negative children with underlying diseases and those without underlying diseases. Boys and children from rural areas represented most cases of pediatric CM. Children exposed to birds/bird droppings or saprophytes and children who did not receive prompt medical attention were at high risk for CM, whereas these factors were not associated with risk for other types of meningitis. These findings suggest that avoidance of environmental risk factors and prompt medical attention are important for the prevention and management of CM among children. Clinicians should consider CM as a potential cause for pediatric meningitis in children with factors mentioned above. Further studies are needed to identify the most effective measures to confront CM.

## Abbreviations

OR: Odds ratio; CI: Confidence interval.

## Competing interests

The authors declare they have no conflicts of interest.

## Authors’ contributions

JG, JZ, XZ, SQ, and YS conceived the study and participated in its design. JG, JZ, SZ, and JL participated in coordination and collected data. XZ, SQ, and YS carried out the laboratory tests. JG and SZ analyzed the data. JG and JZ wrote the manuscript. JG and JL audited AMES database regularly. All authors read and approved the final manuscript.

## Pre-publication history

The pre-publication history for this paper can be accessed here:

http://www.biomedcentral.com/1471-2334/12/376/prepub
